# Molecular and electrophysiological properties underlying calcium signaling in muscle cells of ctenophores

**DOI:** 10.1186/s12915-026-02705-5

**Published:** 2026-08-13

**Authors:** Yuriy Bobkov, Alexandra M. Vargas, Luis F. Vargas, Urvashi Goswami, Joseph F. Ryan

**Affiliations:** 1 Whitney Laboratory for Marine Bioscience, University of Florida, St. Augustine, FL USA; 2 Department of Biology, University of Florida, Gainesville, FL USA; 3 Department of Computer and Information Science and Engineering, University of Florida, Gainesville, FL USA

**Keywords:** Ctenophore, Muscle cells, Calcium imaging, Single-cell sequencing, Electrophysiology

## Abstract

**Background:**

Recent phylogenetic evidence placing ctenophores as a sister lineage to the rest of the animals has challenged existing views as to the origins of muscles, neurons, and nervous systems. Most of the electrophysiological and morphological data available for ctenophores predate the genomic age. An integrated cellular, genomic, and transcriptomic approach has yet to be employed in ctenophores; accordingly, little is known about how ctenophore cells detect, process, and coordinate internal and external signals.

**Results:**

Here we show that excitable cells of the ctenophore *Mnemiopsis* neuromuscular system express a basic set of voltage-gated channels capable of generating and transmitting action potentials. Activation of those cells and generation of action potentials are accompanied by recurrent transient elevations in cytoplasmic Ca^2+^, allowing calcium imaging and evaluation of the activity of populations of sensory cells, neurons, and muscles. Despite the complex evolutionary histories of many of the channels, receptors, and specification factors involved in the operation and specification of these cell types, ctenophore excitable cells share core physiological and transcriptomic properties with those of cnidarians and bilaterians.

**Conclusions:**

Together, these data support an evolutionary scenario in which the muscle and neural cells of ctenophores, cnidarians, and bilaterians descended from an excitable cell present in the last common ancestor of animals.

**Supplementary Information:**

The online version contains supplementary material available at https://doi.org/10.1186/s12915-026-02705-5.

## Background

Understanding how animals convert incoming sensory cues into reactions is a major thrust in neurobiology. This conversion relies on excitable cells: sensory receptor cells, neurons, and/or neuromuscular cell infrastructure. Understanding the origin and evolution of these fascinating cell types and the signaling mechanisms that they employ is a long-standing pursuit in the field of evolutionary neurobiology [1–5]. Nevertheless, in this realm, only modest progress has been achieved over the years compared to other facets of evolutionary biology (e.g., evodevo).

**Fig. 1:**
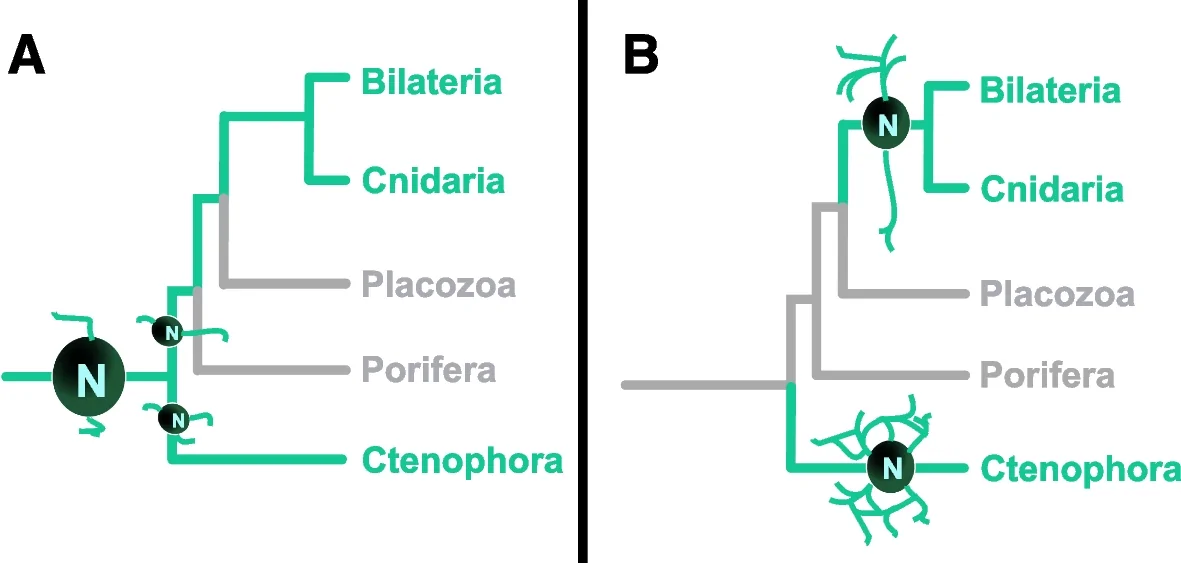
Evolutionary scenarios for the origin of excitable cells in animals. As the sister group to the rest of animals, the ctenophore lineage is important for reconstructing the last common animal ancestor. **A** One scenario places the origin of excitable cell types prior to the last common animal ancestor. **B** The alternative scenario states that excitable cell types arose independently in ctenophores and other animals

Recent phylogenetic evidence placing ctenophores (Fig. 1A) as a sister group to the rest of animals (Fig. 1B; [6–8]) has challenged existing views as to the origins of neurons and in the process increased interest in the evolutionary origin of nervous systems (e.g., [9–14]). In particular, the shifting phylogenetic landscape has led to a debate centering around whether the neurons and muscles of ctenophores arose independently from other animals and were absent from the last common ancestor of animals (e.g., [15]), or if the last common ancestor of animals possessed these excitable cell types, which were subsequently lost or are unrecognizable in sponges and placozoans [13].

Importantly, there is a lack of comprehensive functional information on excitable cells in ctenophores and this gap hinders efforts to the origins and early evolution of animal neural and muscular systems. Pioneering descriptions of the ctenophore neuromuscular system can be traced back to the last quarter of the nineteenth century [16–18]. Since then, multiple generations of ctenophore neurobiologists have conducted meticulous investigations of their own (e.g., [19–23]). Unfortunately, most of the electrophysiological and neuroanatomical data collected on ctenophore sensory and neuromuscular systems were accumulated prior to the genomic age (e.g., [24–26], among many other remarkable studies; see [27] and [28] for review) and lack a corresponding molecular counterpart.

Efforts to provide a morphological and molecular description of the ctenophore neuromuscular system have continued to this day. Recent efforts have involved sophisticated immunohistochemical [29–31] and in situ hybridization experiments [32], supported by global single-cell transcriptome analyses [33], and high-resolution 3D reconstruction using serial block-face scanning electron microscopy [34, 35]. Still, most modern efforts fail to incorporate an integrated functional and physiological perspective in their analyses.

The existing profound disconnect between functional neurophysiological studies and gene expression analyses makes it difficult to develop a high-resolution view of excitable cells and hinders our understanding of the evolution of excitable cells in animals. To address this knowledge gap, we investigate the key molecular components and electrophysiological properties of muscle cells from the ctenophore *Mnemiopsis*. Like neurons, muscle cells also exhibit intrinsic excitability characterized by the generation of action potentials, which enable rapid and effective signal transmission and coordination. Furthermore, both cell types are capable of intercellular signal reception and transmission via chemical and electrical synapses. Activation and action potential generation in both neurons and muscle cells are often accompanied by transient elevations in cytoplasmic Ca^2+^ levels, making calcium imaging a valuable tool for assessing the activity of individual cells as well as cell populations.

Unlike neurons, ctenophore muscle cells are readily available, identifiable, and have been isolated and sustained in primary culture [36–38]. Notably, *Mnemiopsis* possesses a limited repertoire of depolarizing voltage-gated channels—two voltage-gated sodium channels and one voltage-gated calcium channel [39, 40]—as well as a reduced set of other signaling components. This suggests that a common molecular toolkit underpins the functional characteristics shared by all excitable cells in *Mnemiopsis*.

By leveraging the experimental and conceptual accessibility of *Mnemiopsis* muscle cells, we gain new insights into the fundamental mechanisms of excitable cell function in ctenophores. This lays groundwork for future studies aimed at understanding the mechanisms of network-level interactions and the functional and computational capabilities of the ctenophore nervous system. Given the position of ctenophores on the animal tree of life, these data collectively help illuminate the nature of excitable cells in the last common ancestor of all animals.

## Results

### Voltage-gated currents in *Mnemiopsis* muscle cells

**Fig. 2:**
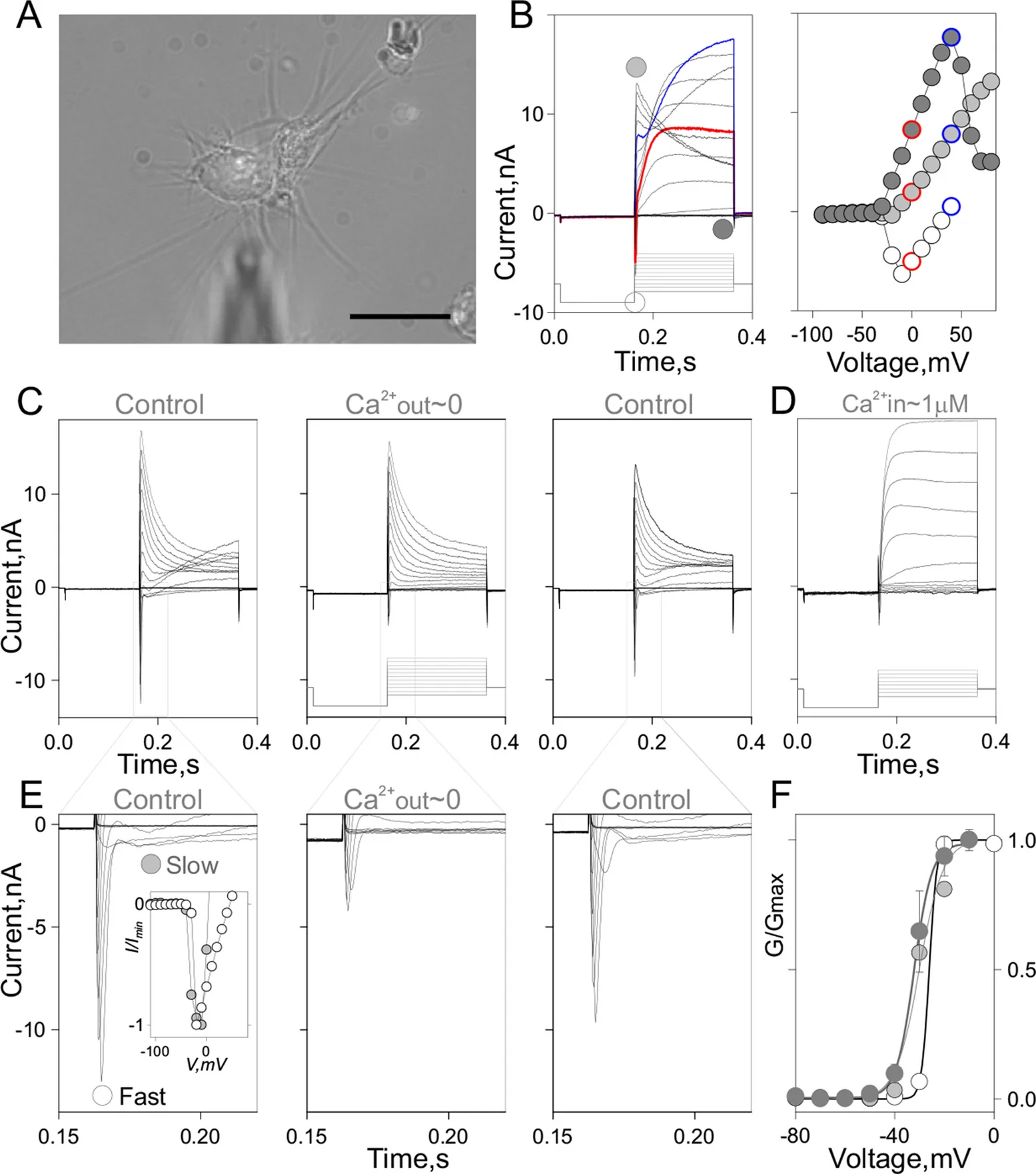
General characteristics of *Mnemiopsis* muscle cell conductances. **A** Typical morphology of muscle cell/muscle cell fragment/s in *Mnemiopsis* primary cell culture. Recording electrode targeting the cell is out of focus (“shadow” at the bottom of the microphotograph). Scale bar = 20 mm. **B** Typical set of voltage-dependent currents recorded from isolated muscle cells: inward currents characterized by fast activation/inactivation kinetics (white circle) likely represent activity of voltage-gated sodium channels (likely gene/s specifications). Fast-activating outward currents (A-type currents, gray circle) are presumably mediated by the activity of delayed rectifier Shak subfamily potassium channels. And, relatively slowly activating outward currents (dark gray circle) presumably mediated by the activity of voltage-gated, calcium-sensitive potassium channels (potentially a broad variety of channel types/genes could contribute to the generation of the current/s, ML04056a, ML128229a, ML01811a). **B** Right panel—respective current–voltage characteristics. Current scales in **B** are the same. The aligned and equally scaled two-panel presentation of current traces and current–voltage (*I*–*V*) relationships allows reliable reconstruction of individual current traces, illustrated here using a color code to selected traces and matching symbols in the *I*–*V* curves. Note, relative ratio of the currents and their kinetic parameters can vary between individual cells (e.g., compare **B** and **C**). **C** Dissection of calcium-dependent components of the integral whole-cell currents. In many cells, we could detect depolarization activated currents characterized by low amplitude and relatively slow kinetics (**E**, gray circle) likely reflecting activity of voltage-gated calcium channels (gene specifications). Removing extracellular calcium reversibly eliminates slow outward currents, increases unknown leak currents, and greatly reduces both fast and slow components of the inward currents (marked by white and gray circles, respectively, **E**). **D** Intracellular calcium greatly increases the amplitude of the outward currents and significantly accelerates the activation kinetics supporting the notion that these currents reflect the activity of the voltage-gated calcium-sensitive potassium channels. Time and current scales in **C** and **D** are the same. Number of depolarizing steps in **D** was reduced as compared to **B** and **C**. **E** Expended view of the same current traces as in **C** showing voltage-gated inward currents. Inset—current–voltage characteristics of fast (white) and slow (gray) inward currents. Note, presumably voltage-gated calcium channels (slower component) are characterized by relatively lower voltage activation threshold. **F** Voltage dependences of inward currents. Conductance values were calculated based on current–voltage characteristics in **E**, *G* = *I*/(*V* − *V*_r_), where *I*—sodium/calcium current peak, *V*—command voltage, and *V*_r_—reversal potentials (*V*_r_NaCh ~ 43 ± 2 mV, estimated experimentally, *n* = 9; *V*_r_CaCh was assumed to be 100 mV). Normalized conductance values (*G*/*G*_max_) were plotted against pulse potential. Boltzmann function fits (smooth lines) for the cell shown here yielded *V*_1/2_s: − 31.6 mV and − 26.1 mV for calcium and sodium activation dependences, respectively. The average *V*_1/2_ for the presumed voltage-gated calcium channels was estimated at − 32.2 ± 0.2 (*n* = 3, dark gray symbols and bars) vs − 22.1 ± 0.4 mV for voltage-gated sodium channels (*n* = 3, analyzed below). To activate currents, the muscle cells were initially hyperpolarized by − 40 to − 50 mV and then 200 ms voltage steps were applied in 10 mV increment (see voltage protocol diagram). Holding potential − 70 mV. Experimental conditions **B**, **C**, **E**—extracellular solution (in mM): NaCl 486, KCl 5, CaCl_2_ 13.6, MgCl_2_ 9.8, Hepes 10 and pH adjusted to 7.8 with NaOH. Intracellular solution: KCl 210, glucose 696, EGTA 1, Hepes 10 and pH adjusted to 7.8 with NaOH. Experimental conditions **D**—extracellular solution (in mM): NaCl 486, KCl 5, CaCl_2_ 13.6, MgCl_2_ 9.8, Hepes 10 and pH adjusted to 7.8 with NaOH. Intracellular solution: KCl 210, glucose 696, CaCl_2_ 0.9 (~ 140 nM Ca^2+^_free_), EGTA 1, Hepes 10 and pH adjusted to 7.7 with Tris-base. Experimental conditions **C**, **E** middle panels—extracellular solution (in mM): NaCl 486, KCl 5, MgCl_2_ 23.4, EGTA 0.1, Hepes 10 and pH adjusted to 7.8 with NaOH. Intracellular solution: KCl 210, glucose 696, EGTA 1, Hepes 10 and pH adjusted to 7.8 with NaOH

We initially characterized the most general electrophysiological properties of *Mnemiopsis* muscle cells using both whole-cell and loose patch extracellular recordings. Isolated muscle cells are usually abundant in primary cell culture [37, 38] and easily identifiable both morphologically and functionally (Fig. 2; Additional file 1: Video 1). They are also sensitive to potential transmitters, including glutamate (Additional file 2: Fig. S1, Additional file 3: Video S1; [15, 38, 41, 42]), and capable of generating action potentials in a highly consistent, concentration-dependent manner [15].

We analyzed whole-cell currents using patch-clamp recordings in voltage-clamp mode. Voltage-activated currents were initially characterized by applying standard voltage-step protocols. Under these experimental conditions, membrane hyperpolarization of the *Mnemiopsis* muscle cells did not evoke any substantial currents, whereas depolarization triggered the activation of several types of voltage-gated channels, including sodium and calcium channels (Fig. 2E and F) along with a variety of potassium channels. Among potassium channels, the delayed rectifier potassium channel subfamily is likely to mediate fast-activating outward currents (known as A-type currents, Fig. 2C), while calcium-sensitive potassium channels presumably mediate the more slowly activating outward currents (Fig. 2D).

The activity of excitable cells, particularly muscle cells, depends on calcium, which functions both as a charge carrier and as an important regulator of ion channel activity and downstream signaling pathways. We therefore examined the overall effects of calcium on cell conductances. Reducing extracellular calcium concentration (by omitting added calcium and substituting with magnesium to maintain ionic strength in artificial sea water) reversibly decreased the amplitude of inward currents mediated by fast-activating voltage-gated sodium channels and eliminated putative calcium currents. These effects suggest that calcium serves as a charge carrier for both sodium and calcium channels. The reversible reduction in the amplitude of fast-activating/inactivating voltage-gated sodium channels (Fig. 2C–E) may be explained by the sodium channel-conducting pore not significantly discriminating between calcium and sodium ions.

In addition, the use of calcium-free artificial sea water reversibly decreased the amplitude of slow outward currents, which are likely mediated by voltage-gated calcium-activated potassium channels. Increasing intracellular calcium by direct application through the recording pipette significantly enhanced the amplitude of these currents and accelerated their activation kinetics, supporting the identification of these channels as voltage-gated calcium-sensitive potassium channels (Fig. 2D).

Comparison of voltage-dependent activation showed that the voltage-gated calcium channels were activated at more hyperpolarized (negative) voltages than the voltage-gated sodium channels (− 32 vs − 26 mV, for the cell shown in Fig. 2). To better characterize these channels, a thorough pharmacological approach is required to selectively isolate and study the relatively minor voltage-gated calcium channel component.

In summary, although the relative ratios of the currents and their kinetic parameters may vary significantly between individual cells (e.g., Fig. 2B vs Fig. 2C), isolated *Mnemiopsis* muscle cells are characterized by a set of voltage-gated channels, which, in cnidarians and bilaterians, are crucial for generating action potentials and commonly expressed in excitable cells, including neurons.

### Functional properties of *Mnemiopsis* voltage-gated Na + channels

**Fig. 3:**
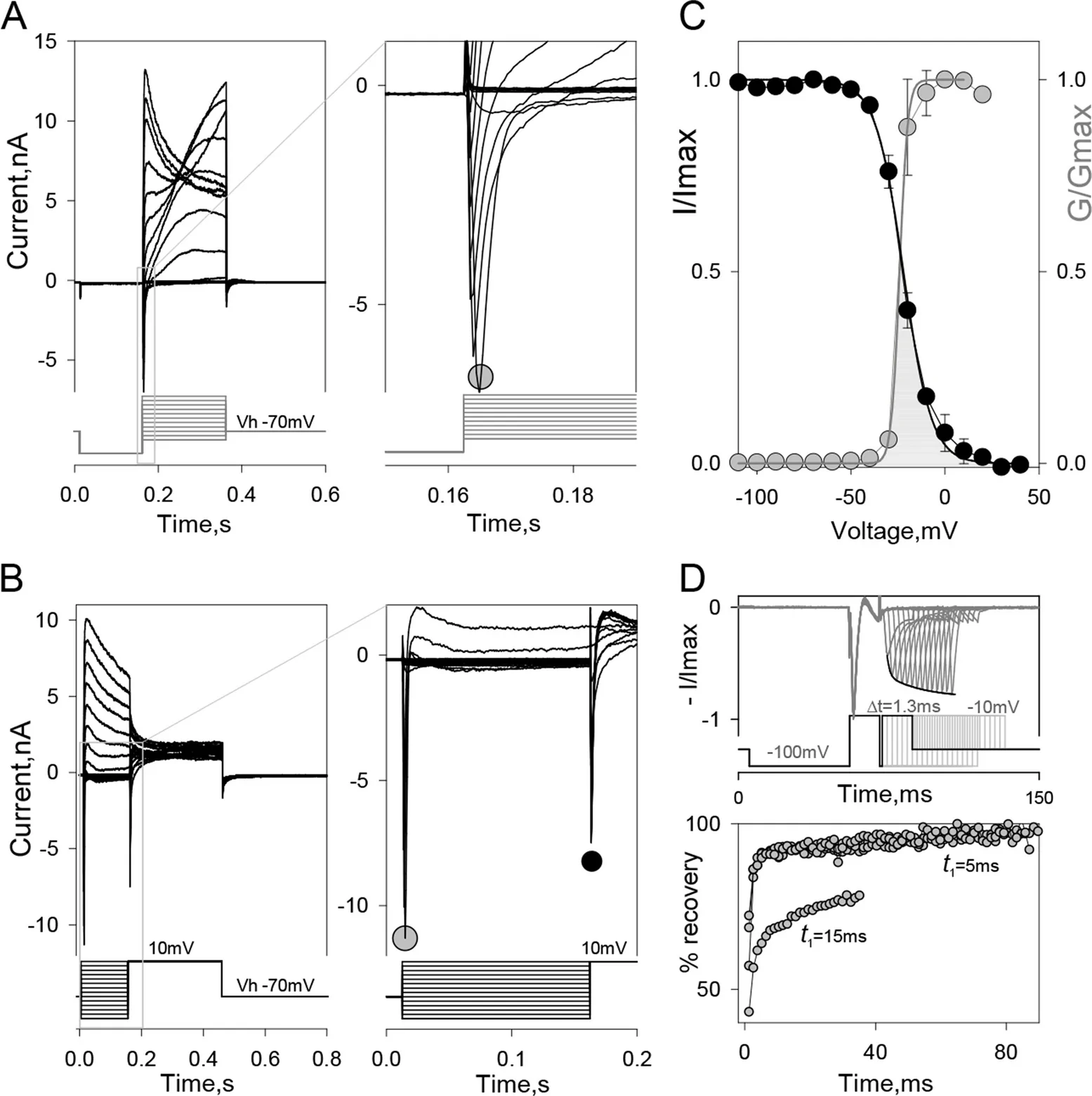
Basic parameters of voltage-dependent sodium channels recorded from *Mnemiopsis* muscle cells. Activation, inactivation, recovery from inactivation. **A** Activation. Representative whole-cell currents activated by depolarization. Right panel shows currents carried by voltage-gated sodium channels on an extended time/current scales. To activate currents, the muscle cell was initially hyperpolarized to − 110–120 mV for 150 ms and then 200 ms voltage steps were applied in 10 mV increment (see voltage protocol diagrams). Peak current values (gray circles) were used to calculate *G*/*G*_max_ and generate the activation curve (**C**). **B** Inactivation. Representative whole-cell currents obtained using steady-state inactivation protocol. To induce fast inactivation of the sodium channels, 150 ms voltage steps were applied from a holding potential of − 70 mV to membrane potentials ranging from − 120 to + 10 mV in 10 mV increment (as shown on diagrams at the bottom of current curves plots). Then cells were depolarized by a test pulse to − 10 ± 10 mV to estimate the fraction of still active channels (black circles) and generate steady-state inactivation curve (**C**). **C** Voltage-gated sodium channel activation (gray) and inactivation (black) dependences generated using protocols described in **A**, **B**. Conductance (*G*) was determined as *G* = *I*/(*V* − *V*_r_), where *I*—sodium current peak, *V*—command voltage, and *V*_r_—reversal potential (43 ± 2 mV, estimated experimentally, *n* = 9). Normalized current values (*I*/*I*_max_) were plotted against prepulse potential. Boltzmann function fit (smooth lines) yields *V*_1/2_ s: − 24.2 ± 0.2 mV (*n* = 6) and − 22.1 ± 0.4 mV (*n* = 3) for inactivation and activation dependences, respectively. **D** Recovery from inactivation of currents carried by voltage-gated sodium channels. Representative whole-cell current traces elicited by standard double-pulse protocol (top panel): during each sweep, the first pulse (15 ms duration) depolarized the membrane from − 100 to − 10 mV and induced a transient inactivating inward current. A second identical pulse was applied Δt*n ms after the first voltage step, where Δ*t* is 1.3 ms increment and *n* is a sweep number. Current traces were individually normalized to the peak amplitude of the current elicited by the first voltage step of each sweep. Bottom panel—percentage recovery from inactivation. Note, currents recover from inactivation faster if the duration of the first depolarizing step is shorter. Data were approximated by *f*(*t*) = *a*(1 − exp(− *bt*)) (smooth lines) with *t* ~ 3.3 and ~ 17.6 ms for 5 ms and 15 ms, respectively. Data shown in **D** were collected from the same cell. Note, **D** is intended to be an example of the recording configuration: excitation voltage protocol and current recovery profile, rather than a quantitative summary across the population. These experiments are technically more demanding and require prolonged voltage protocols, limiting the number of stable recordings. Nevertheless, while illustrated here for a single cell, the observed overall properties and temporal characteristics of recovery from inactivation are consistent with those reported for sodium currents. Further optimization of experimental conditions would facilitate more systematic characterization of recovery from inactivation and enable comparison of these kinetic parameters across cell subpopulations. Experimental conditions—extracellular solution (in mM): NaCl 486, KCl 5, CaCl_2_ 13.6, MgCl_2_ 9.8, Hepes 10 and pH adjusted to 7.8 with NaOH. Intracellular solution: KCl 210, glucose 696, EGTA 0.5–1, Hepes 10 and pH adjusted to 7.8 with NaOH

To characterize the voltage-dependent activation of voltage-gated sodium channels (VGSCs), we applied depolarizing voltage steps in 10 mV increments to elicit channel activation (Fig. 3). We used peak current values (Fig. 3A, right panel) to calculate normalized conductance (*G*/*G*_max_) and to construct a sodium channel activation curve (Fig. 3B, C, gray symbols). Fitting a Boltzmann function to the final dataset (mean values, *n* = 6) yielded a half-maximum activation (*V*_1/2_) of approximately − 24 mV and slope factor (*k*) of ~ 0.46. Using the relationship *k* = *Z*_d_*F*/*RT*, we estimated a gating charge valency (*Z*_d_) of ~ 7.8.

To obtain parameters of voltage-dependent steady-state inactivation of *Mnemiopsis* VGSCs, 150-ms-long prepulses were applied to potentials ranging from − 120 to 10 mV, in 10 mV steps, followed by a test pulse to 10 mV (Fig. 3B). The peak amplitude of the currents evoked by the test pulses was normalized to the maximal currents and plotted as a function of the prepulse potential (Fig. 3C). Fitting of the Boltzmann distribution (descending form) to the final dataset (mean values, *n* = 3, Fig. 3C, smooth line) yielded a half-maximum inactivation value (*V*_1/2_) of approximately − 22 mV and a slope of the fit (*k*) of ~ 0.13.

Overlaying the steady-state inactivation and activation curves of *Mnemiopsis* VGSCs revealed an unusually pronounced overlap (i.e., window current range) indicating that a substantial fraction of VGSCs remain functional at membrane potentials well within the depolarization range (Fig. 3C, shaded area). The functional role of the *Mnemiopsis* sodium channel window current range is uncertain, but it likely contributes to establishing the resting membrane potential of *Mnemiopsis* excitable cells.

To characterize the recovery of *Mnemiopsis* VGSCs from fast inactivation, we used the following pulse protocol (Fig. 3D): voltage clamping at − 70 mV holding potential, membrane hyperpolarization to − 100 mV for 40 ms to remove possible residual inactivation, and then a conditioning depolarizing 5-ms voltage pulse to − 10 mV, which activated an inward Na^+^ current that exhibited fast inactivation (Fig. 3D), and then a second identical pulse 1.3 ms later. The process was repeated with the time between pulses gradually increased from 1.3 to 87.1 ms.

Finally, the fractional recovery of Na^+^ channels from inactivation was calculated and plotted as a function of the time between successful voltage steps (Fig. 3D, lower panel). This recovery was best approximated with a double exponential function (*t*_1_ ~ 0.9, *t*_2_ ~ 29 ms), as shown for a representative cell (Fig. 3D). The second, slower time constant seems to be more prominent following longer depolarizing pulses (Fig. 3D, 15 ms; for the cell shown, the recovery was not complete).

The electrophysiological properties of *Mnemiopsis* VGSCs are broadly consistent with previously characterized voltage-gated sodium channels from other species under various experimental conditions and ion environments. For example, like the VGSCs of non-ctenophores, *Mnemiopsis* VGSCs rapidly activate and inactivate, and have an activation threshold between − 60 and − 40 mV.

### Functional properties of voltage-gated K + channels in *Mnemiopsis* muscle cells

**Fig. 4:**
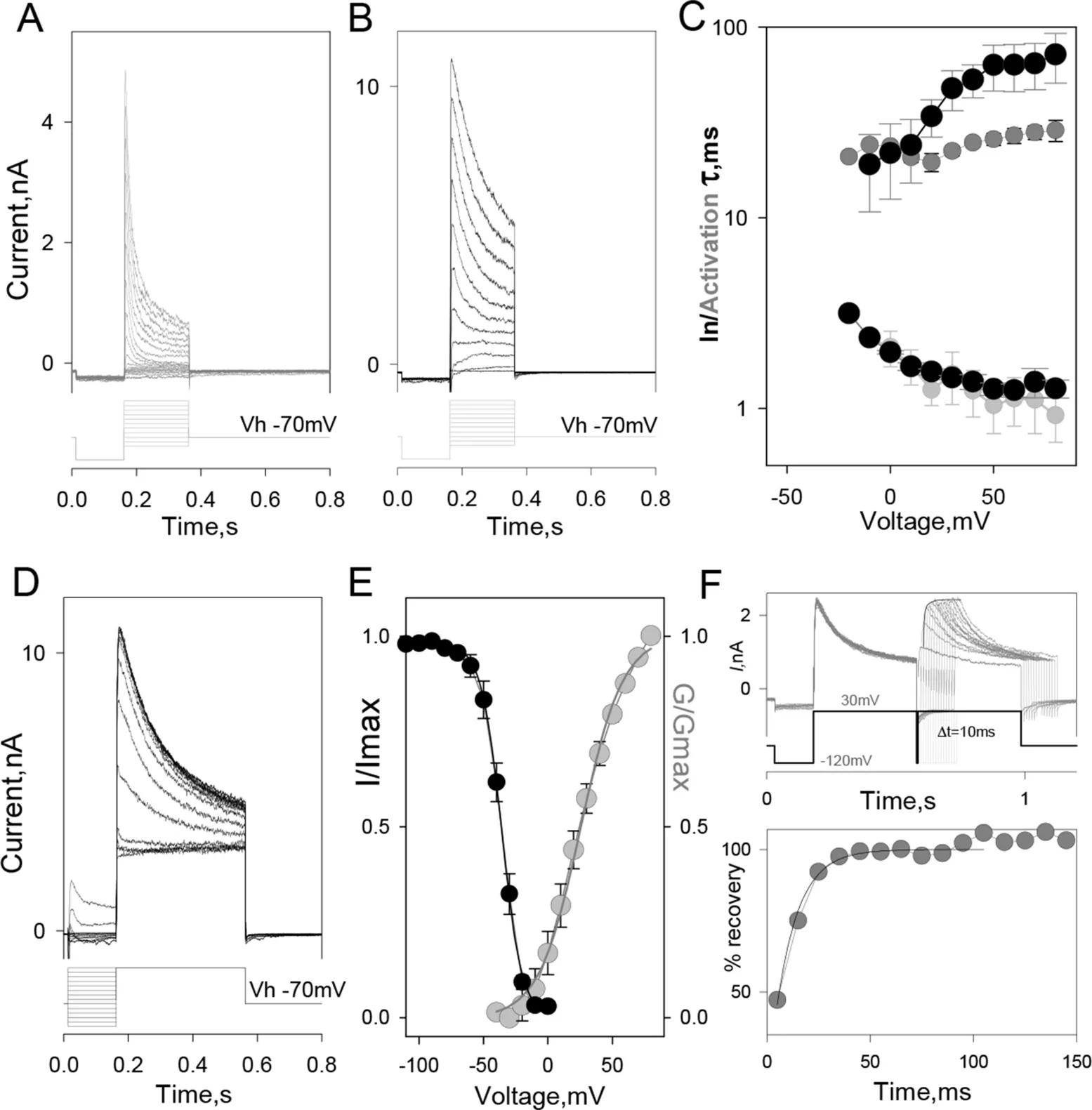
Basic parameters of fast inactivating, presumably A-type potassium currents, recorded from *Mnemiopsis* muscle cells. Activation, inactivation, recovery from inactivation. **A**, **B** Voltage-dependent activation of transient outward currents. Currents were evoked by 200 ms depolarizing steps from − 100 mV to + 80 mV with 10 mV increment, following a 150-ms conditioning prepulse to − 110 mV (diagram below family of the current traces). The current recordings obtained applying the activation protocol were used to extract current kinetic parameters (**C**) and analyze voltage dependence of activation (**D**, **E**). **C** Voltage dependence of activation/inactivation kinetics. Current traces characterized by well-pronounced A-type current component were low-pass filtered at 1.5 kHz, individually normalized with respect to peak current amplitude and fit with *I*(*t*) = *I*_max_(1 − exp(− *t*/*t*_r_))*exp(− *t*/*t*_d_) + *c* to estimate current rise (*t*_r_, activation) and decay (*t*_d_, inactivation) time constants. Data points represent mean values ± SE (*n* = 4–7). Note **C** is a semi-logarithmic plot. **D** Voltage-dependent inactivation of transient outward currents. To analyze inactivation of the transient potassium channels, 150 ms voltage steps were applied from a holding potential of − 70 mV to membrane potentials ranging from − 120 to + 10 mV in 10 mV increments (as shown on diagrams at the bottom of current curves plots). Then cells were depolarized by 400 ms test pulse to + 80 mV to estimate the fraction of still active channels (black circles) and generate steady-state inactivation curve (**E**). Intracellular solution contained 1 mM EGTA to minimize activity of calcium-sensitive voltage-gated potassium channels. Note, no pharmacological agents were used to isolate the transient potassium currents. Cells chosen for the analysis had predominant A-type potassium currents, so other potassium channels, if any, were ignored. **E** Voltage dependences of activation (gray circles) and inactivation (black circles) of A-type like potassium currents. Data obtained in experiments described in **A** were used to generate activation curve. Peak current amplitudes (*I*_peak_) were determined at each voltage step and used to calculate conductance: *G*(*V*) = *I*_peak_/(*V* − *V*_rev_), where *V* is the test potential and *V*_rev_ is the potassium current reversal potential (− 94 mV) estimated for given potassium ion gradient. The conductance values were normalized to the maximal conductance (*G*_max_) of each cell (total number of cells analyzed = 4) and the averaged conductance values were plotted as a function of *V*. Data obtained in the experiments described in **D** were used to generate voltage dependence of steady-state A-current inactivation. Peak current amplitudes were determined for each cell, normalized to *I*_max_ and averaged. Fitting activation *G*(*V*) and inactivation *I*(*V*) curves with different forms of the Boltzmann equation (*Y* = 1/[1 + exp(*k*(*V* − *V*_1/2_))] or *Y* = 1/[1 + exp(*k*(*V*_1/2_ − *V*))]) yielded *V*_1/2_ s: 22.7 ± 0.8 and − 36.1 ± 0.6 mV, respectively. **F** Recovery from inactivation of calcium-independent potassium A-currents. A-type currents were elicited by double-pulse protocol: the first conditioning pulse (400 ms duration) depolarized the membrane from − 120 to 30 mV and induced inactivation of A-current. A second identical pulse was applied Δt*n ms after first voltage step, where Δ*t* is 10 ms increment and *n*—sweep number. The membrane was hyperpolarized to − 120 mV during recovery (interpulse) interval. Current traces were individually normalized to the peak amplitude of the current elicited by the first voltage step of each sweep. Bottom panel: percentage recovery from inactivation. Data were approximated by *f*(*t*) = *a*(1 − exp(− *bt*)) (smooth line) with *t* ~ 11 ms. Prepulse was − 120 mV. Note that **F** is intended to illustrate the recording configuration (i.e., excitation voltage protocol and current recovery profile) and is not intended as a quantitative summary across the population. Although illustrated here for a single cell, the observed properties and recovery from inactivation fall within the range reported for typical A-currents. Experimental conditions—extracellular solution (in mM): NaCl 486, KCl 5, CaCl_2_ 13.6, MgCl_2_ 9.8, Hepes 10 and pH adjusted to 7.8 with NaOH. Intracellular solution: KCl 210, glucose 696, Ca^2+^ 0.9, EGTA 1, Hepes 10 and pH adjusted to 7.8 with NaOH

We next characterized the properties of voltage-gated transient potassium current channels (A-type potassium channels). The extensive diversity reported for channels of this type [43–45] suggests a wide range of functional phenotypes, which is at least partially consistent with our findings. We observed that activation and inactivation kinetics of transient potassium currents varied among individual muscle cells (Figs. 2 and 4). Although A-type potassium currents were not pharmacologically isolated, we included 1 mM EGTA in the intracellular solution to minimize calcium-activated voltage-gated potassium channel activity. Additionally, the cells chosen for the analysis exhibited predominant A-type outward potassium currents, and hence other potassium currents and channel types, if any, were ignored (Fig. 4A and B). To estimate kinetic parameters of activation/inactivation, A-type potassium currents were evoked by 200 ms depolarizing steps from − 100 mV to + 80 mV with 10 mV increment, where each step was preceded by a 150-ms conditioning prepulse to − 110 mV to remove possible residual inactivation (Fig. 4A and B). Current traces characterized by a well-pronounced A-type current component were individually normalized with respect to peak current amplitude and fit with a double exponential function to estimate current rise (*t*_r_, activation) and decay (*t*_d_, inactivation) time constants. Data clearly clustered in two groups (Fig. 4A–C).

Current traces were processed identically across cells, including offline low-pass filtering at 1.5 kHz (*t*_filter_ ~ 0.1 ms) and normalization. Fitted time constants were used for relative comparison between fast- and slow-inactivating subpopulations. For fast-inactivating cells, *t*_r_s exhibited voltage dependence, decreasing from 2.33 ± 0.03 ms at − 10 mV to approximately 1.0–1.2 ms between + 50 and + 70 mV, and reaching 0.92 ± 0.26 ms at + 80 mV. In slow-inactivating cells, *t*_r_s ranged from 2.35 ms at − 10 mV to 1.24 ± 0.10 ms at + 60 mV, with values remaining near ~ 1.3 ms at more depolarized voltages. Overall, both subpopulations exhibited rather similar activation kinetics within this voltage range.

Inactivation kinetics were noticeably different between subpopulations. In fast-inactivating cells, inactivation time constants showed relatively weak voltage dependence, falling within the range of ~ 20–29 ms across voltages (e.g., 24.1 ± 0.6 ms at − 10 mV and 28.8 ± 3.7 ms at + 80 mV). In contrast, slow-inactivating cells exhibited substantially slower and strongly voltage-dependent inactivation, with time constants increasing from ~ 19–24 ms at − 10 to + 10 mV to ~ 63–72 ms at + 50 to + 80 mV. Different approaches, including pharmacological channel isolation, would be required for more accurate estimation of intrinsic channel kinetics and their interpretation.

Since currents characterized by slower kinetic parameters predominated in 12 cells (vs 5 cells), those cells were used for further analysis of activation and inactivation voltage dependence. Peak current amplitudes were determined at each voltage step and used to calculate conductance with the equation *G*(*V*) = *I*_peak_/(*V* − *V*_rev_), where *V* is the test potential and *V*_rev_ the potassium current reversal potential (− 94 mV) estimated for a given potassium ion gradient. Conductance values were normalized to the maximal conductance (*G*_max_) of each cell, and the averaged values were plotted as a function of *V* (Fig. 4E).

We analyzed the inactivation of transient potassium channels using a standard two-pulse steady-state inactivation protocol. Starting from a holding potential of − 70 mV, we applied voltage steps to membrane potentials ranging from − 120 to + 10 mV in 10 mV increments (Fig. 4D). We then depolarized the cells to + 80 mV to assess the fraction of channels that remained active (Fig. 4E). For each cell, we measured peak current amplitudes, normalized to *I*_max_, and averaged the values. We plotted the results as a function of the prepulse potential (Fig. 4E). We fitted the activation and inactivation curves with Boltzmann equations, which yielded half-maximum voltage (*V*_1/2_) values of 22.7 ± 0.8 mV for *G*(*V*) and − 36.1 ± 0.6 mV for *I*(*V*) (mean ± SEM, *n* = 4).

As with other channel types, we assessed A-current recovery from inactivation using a double-pulse protocol. We applied a 400-ms conditioning pulse that depolarized the membrane from − 120 to 30 mV, inducing A-current inactivation. We then delivered a second identical pulse, initially separated by a 10-ms interval. We repeated this protocol while gradually increasing the interval between pulses from 10 to 150 ms. During the interpulse recovery interval, we hyperpolarized the membrane to − 120 mV. We normalized each current trace to the peak amplitude of the current elicited by the first voltage step in the sweep. We then plotted the fractional A-current recovery from inactivation as a function of time between conditioning and testing voltage pulses. We approximated the resulting curve with the function *f*(*t*) = *a*(1 − exp(− *bt*)) (a smooth line) and estimated a recovery time constant (*t*_rec_) of approximately 18 ms at − 120 mV.

While potential molecular correlates of the *Mnemiopsis* A-type potassium currents have been genetically identified [43], their direct association with a specific functional phenotype has yet to be established [44, 45]. However, the *Mnemiopsis* A-currents appear to be similar to Shaker family members in all major functional characteristics (kinetics, voltage dependencies, recovery from inactivation).

### Calcium signal correlates with activity of *Mnemiopsis* muscle cells

Calcium ions function as fundamental cytoplasmic messengers in virtually every cell type across all forms of life. Consequently, analyzing the spatiotemporal dynamics of calcium provides powerful insights into the behavior of individual cells and the emergent properties of excitable cell networks.

To establish real-time monitoring of intracellular calcium dynamics in live *Mnemiopsis* cells, we optimized the use of conventional membrane-permeable calcium-sensitive (acetoxymethyl (AM) neutralized) fluorescent probes (both Fluo-4 AM and Calbryte-520 AM). We developed a simple method to greatly improve the loading of *Mnemiopsis* cells with such probes. Using primary cell culture preparation that maintains viability of the *Mnemiopsis* cells for many hours, we began by identifying and characterizing the spontaneous and evoked activity of different cell types (Additional file 1: Video 1; Additional file 7: Video 2; Additional file 8: Video 3). This single-cell approach offers exceptional opportunities for studying calcium dynamics and the molecular mechanisms of signal transduction in great detail. More importantly, it enables us to address fundamental yet often overlooked questions, such as whether, and to what extent, intracellular calcium signals accurately reflect the electrical activity of *Mnemiopsis* excitable cells.

**Fig. 5:**
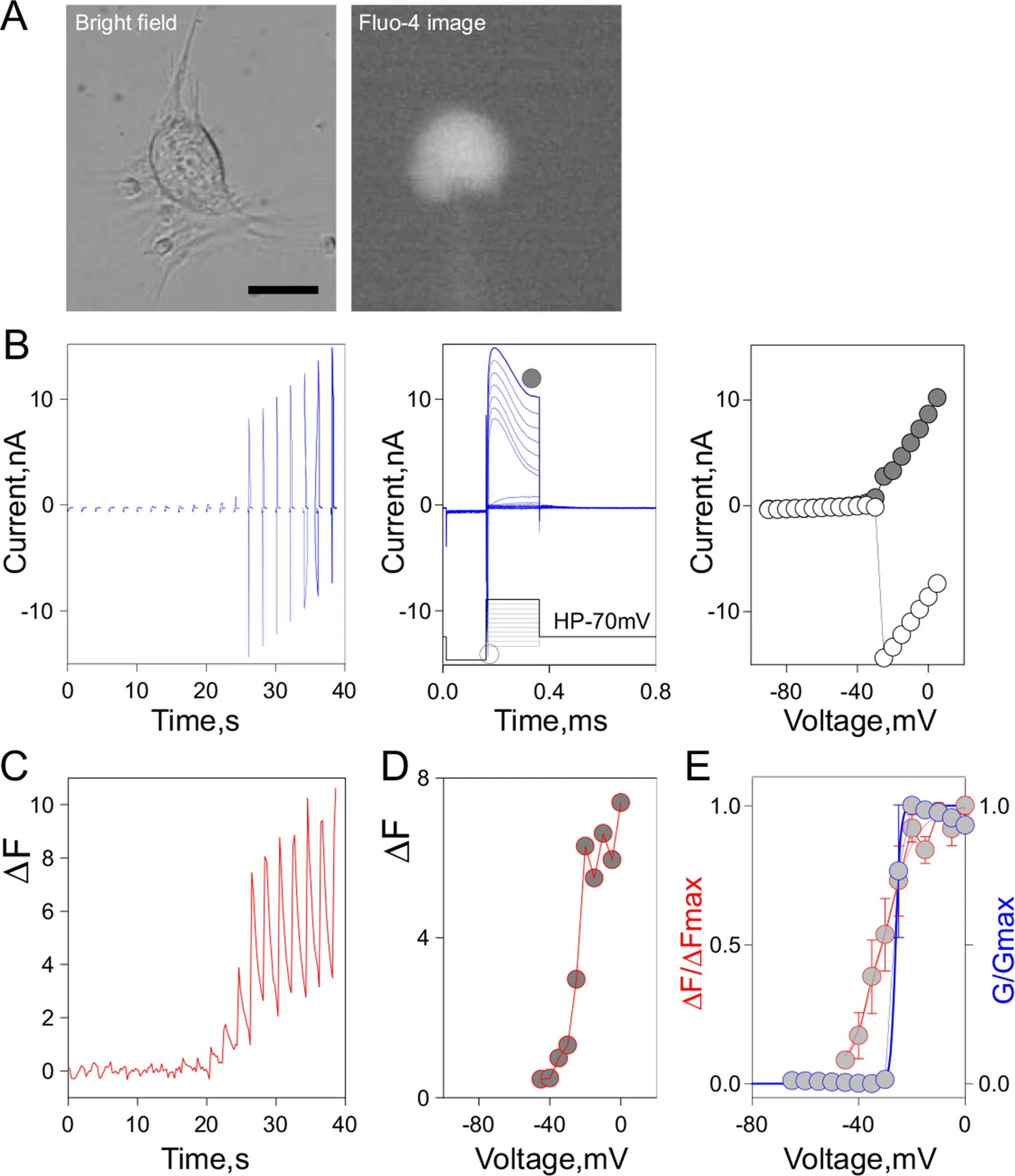
Voltage-dependent calcium signaling in *Mnemiopsis* muscle cells. **A** The cell was dialyzed with calcium-sensitive probe (Fluo-4, ~ 5 mM) through patch pipette. **B** Time course (left), family of whole-cell currents (middle), and current–voltage characteristics of the cell voltage-dependent conductances. To activate currents, the muscle cell was initially hyperpolarized by − 50 mV and then 200 ms voltage steps were applied in 5 mV increment (see voltage protocol diagram in Fig. 1B, middle). *I*/*V* relationships (**B**, right panel) for transient inward component (peak currents, empty circles) and steady-state currents (gray circles), determined at locations marked by corresponding symbols in middle panels. **C** Calcium signal responses to voltage steps aligned relative to current traces (**B**). Note, depolarizing voltages transiently increase intracellular calcium concentration. The calcium signal kinetics and amplitude are voltage-dependent (**C**, **D**). **D** Voltage dependence of calcium signal generated by the cell shown in **A**, **B**, **C**. **E** Voltage dependence of calcium signal appears to be different from voltage dependence of voltage-gated sodium channels (blue circles and line), perhaps, suggesting that a significant portion of the calcium currents is carried by another calcium ion transport system/s, e.g., by voltage-gated calcium channels (still electrophysiologically undetectable in the given ionic conditions for this particular cell). Both the fluorescence intensity and the sodium channel conductance were normalized to maximum values. Data are presented as average Δ*F* (red circles and bars, *n* = 3) and *G*_Na_ (blue circles and bars, *n* = 3). Data in **E** include data shown in **D**. Experimental conditions—extracellular solution (in mM): NaCl 486, KCl 5, CaCl_2_ 13.6, MgCl_2_ 9.8, Hepes 10 and pH adjusted to 7.8 with NaOH. Intracellular solution: KCl 210, GTP 0.5, ATP 0.5, MgCl_2_ 1, glucose 696, EGTA 0.5, Hepes 10 and pH adjusted to 7.8 with NaOH. Current scales are the same in **B**. Voltage scales in **B** (right panel), **D**, and **E** are the same. Scale bar in **A**—20 mm. The amplitude of the calcium response was estimated as Δ*F*_max_ − preceding Δ*F*_min_. So, lack of recovery of the calcium signal to the initial basal level was ignored. Conductance (*G*) was determined as *G* = *I*/(*V* − *V*_r_), where *I*—peak current, *V*—command voltage, and *V*_r_—reversal potential. Fitting activation curves Δ*F*(*V*) and *G*_Na_(*V*) to a Boltzmann function modification (*Y* = 1/[1 + exp(*k*(*V*_m_ − *V*_1/2_))]) yielded *V*_1/2_s: − 28.7 and − 26.2 mV, respectively

To address this question, we first assessed the role of voltage-gated calcium and/or sodium channels in mediating calcium signaling in *Mnemiopsis*. We dialyzed Fluo-4 penta-potassium salt into dissociated muscle cells. We then simultaneously recorded electrophysiological and calcium signals (Fig. 5A–C). To activate voltage-gated muscle cell currents, depolarizing 200 ms voltage steps were applied in 5–10 mV increments (see voltage protocol diagram in Fig. 5B, middle). We observed depolarizing voltages to transiently increase the intracellular calcium concentration in a voltage-dependent manner (Fig. 5C and D). The voltage dependence of calcium signal appears to differ from that of sodium channels (Fig. 5E, blue circles and line), which may suggest that a significant portion of calcium currents are carried by one or more other Ca^2+^ transport systems, such as voltage-gated calcium channels, which are electrophysiologically undetectable in the ionic conditions used for this experiment. Notably, the voltage dependence of calcium signals appears to agree with voltage-dependent activation of the presumed voltage-gated calcium current (slow inward current component, Figs. 2 and 5E).

Further research is needed to fully characterize all aspects of calcium signaling in *Mnemiopsis* excitable cells, including the possible role of calcium immobilization from intracellular stores. Nevertheless, it appears that calcium signals correlate well with the activation of channels typically involved in action potential generation. Thus, calcium imaging is a useful tool for monitoring the electrical activity of *Mnemiopsis* cells.

**Fig. 6:**
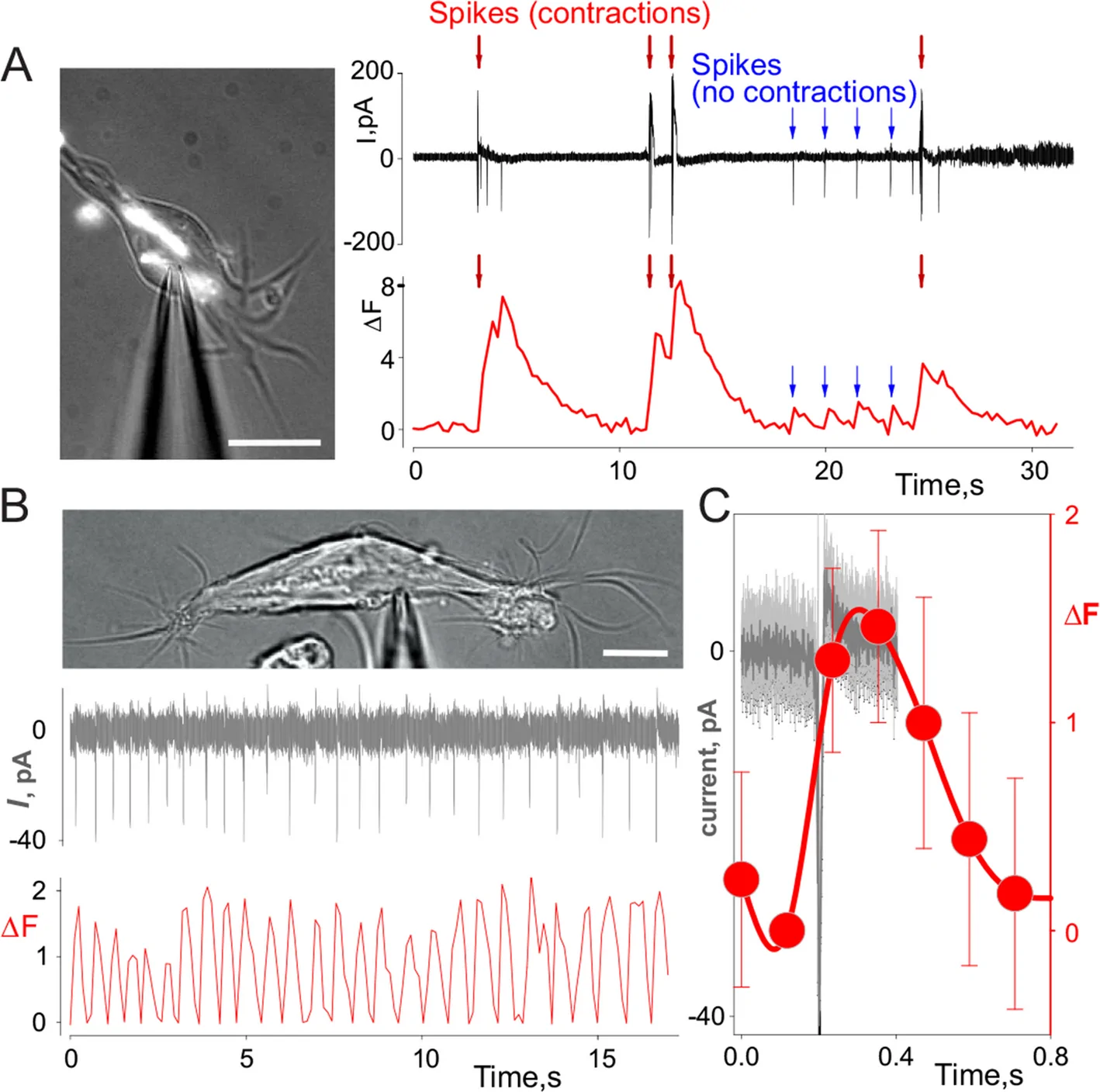
Intracellular calcium signal correlates with the electrophysiological activity of the *Mnemiopsis* muscle cells. Spontaneous activity of different muscle cells in primary culture. Simultaneous extracellular loose patch recording (**A**, **B**, spikes, top traces) and calcium signal (**A**, **B**, red, bottom traces) recordings from individual muscle cells. The activity of the cell in **A** exhibits bimodal activity pattern: tonic activity mode, when individual spikes do not trigger muscle cells contractions (**A**, blue arrows, also cell in **B**), and bursting activity mode, when bursts of action potentials produce greater increase in cytoplasmic calcium concentration high enough to trigger muscle cell contractions (red arrows). **C** Average spike (gray) and average calcium signal oscillation (red) were aligned. Error bars are standard deviations. The cells were loaded with a calcium-sensitive probe (Fluo-4am). Slow changes in fluorescent intensity levels in **A**, **B** mainly caused by calcium indicator bleaching were manually subtracted. Note, the calcium signal reflects the electrophysiological activity of the *Mnemiopsis* muscle cells and can be reliably used to detect even individual action potentials. Scale bars, 20 mm. Experimental conditions—extracellular solution (in mM): NaCl 486, KCl 5, CaCl_2_ 13.6, MgCl_2_ 9.8, Hepes 10 and pH adjusted to 7.8 with NaOH

The next question we aimed to address was whether, and to what extent, the intracellular calcium signal reflects the activity of excitable cells. To investigate this aspect, we employed a minimally disrupting approach in which primary cultured muscle cells were loaded with Fluo-4 and their electrical (spike) activity was simultaneously monitored using extracellular loose patch recording. As shown for a representative cell in Fig. 6A, the spontaneous activity of some cells was characterized by two modes, tonic and bursting. During tonic activity mode, the cells would generate action potentials without contracting or twitching, a behavior phenomenologically consistent with neuronal behavior (Fig. 6A blue arrows and B). In bursting activity mode, bursts of action potentials produced a cytoplasmic calcium concentration high enough to trigger muscle cell contractions (red arrows). Both bursting and spiking events are accompanied by a proportional increase in calcium concentration. Comparison of calcium signal oscillations and spiking dynamics indicates that spike generation occurs later in the calcium oscillation cycle and may even be triggered by calcium increase (Fig. 6A and B, right). This observation would be consistent with the major calcium influx being provided by voltage-gated calcium channels, which may require less depolarization for activation compared to other calcium transport systems, including voltage-gated sodium channels (Figs. 2 and 5). However, it is important to consider the kinetic characteristics of both voltage-gated calcium and sodium channel activation, as these properties can influence calcium response and spiking dynamics. In general, the calcium signal is well correlated with the electrophysiological activity of the excitable cells and can be reliably used to detect various activity patterns, including individual action potentials.

### Transcriptomic profile of individual muscle cells

Targeted single-cell transcriptome analysis (scRNA-Seq) is becoming an indispensable tool for capturing cell type diversity and identifying specific cell types. In this study, we employed scRNA-Seq and transcriptome analysis of identified muscle cells to characterize their molecular profile and establish initial correlations with their biophysical properties. We collected muscle cells using disposable glass pipettes and used the DNBSEQ Platform and Smart-Seq II amplification for single-cell library construction, amplification, and subsequent deep sequencing. Four single-cell samples were collected (Additional file 4: Fig. S2), passed quality control tests, sequenced, and further analyzed.

**Fig. 7:**
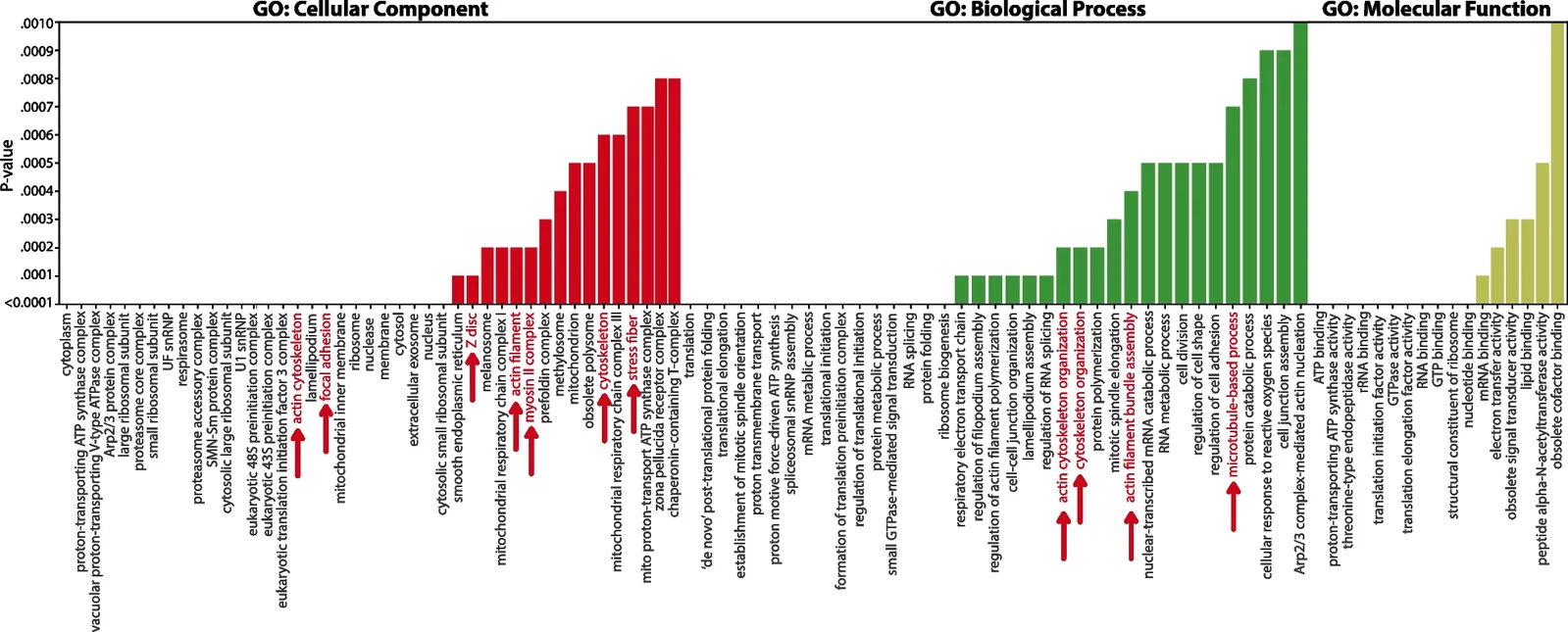
Gene ontology (GO) categories overrepresented in genes expressed in all four *Mnemiopsis* muscle cells (*P* ≤ 0.001). *P* value based on Monte Carlo analysis. GO categories without a bar had a *P* value < 0.0001. GO categories associated with muscle (i.e., actin cytoskeleton, focal adhesion, Z disc, actin filament, myosin II complex, cytoskeleton, stress fiber, actin cytoskeleton organization, cytoskeleton organization, actin filament bundle assembly, and microtubule-based process) are highlighted with arrows. Full list of overrepresented GO categories is in our GitHub repository

We conducted gene ontology (GO) enrichment analysis using Monte Carlo simulations to identify significantly overrepresented GO terms associated with genes that were expressed in all four of our muscle cell transcriptomes. These included several GO categories known to be directly involved in muscle physiology (Fig. 7) including actin cytoskeleton (GO:0015629), focal adhesion (GO:0005925), Z disc (GO:0030018), actin filament (GO:0005884), myosin II complex (GO:0005859), cytoskeleton (GO:0005856), stress fiber (GO:0001725), actin cytoskeleton organization (GO:0030036), cytoskeleton organization (GO:0007010), actin filament bundle assembly (GO:0061572), and microtubule-based process (GO:0007017).

In addition to identifying GO categories that were overrepresented in our data, we compared our data to sets of genes associated with muscle function and specification. First, we identified 47 genes expressed in all four of our muscle cell transcriptomes that belonged to GO categories associated with mouse muscle cells as defined by Raza et al. [46]. This included several immediately recognizable muscle-related genes like actin, tubulin, myosin regulatory light chain, myosin heavy chain, tropomodulin, titan, and several actin binding factors to name a few (Additional file 9: Table S1). We also identified 16 genes coding for ion channels or cell membrane receptors expressed in all four muscle cells including 2 innexins, 3 G-protein coupled receptors, an amiloride-sensitive cation (ASIC) channel, an ionotropic glutamate receptor, and a voltage-gated potassium channel (Additional file 9: Table S2). We detected a voltage-gated sodium or calcium channel expressed in three of the four muscle cells including the voltage-gated sodium channels ML358826a and ML17059a expressed in three and two cells, respectively, and the voltage-gated calcium (Ca_V_2) channel ML190424a expressed in one cell (Additional file 9: Table S2).

We also detected a broad set of developmental factors expressed in all four of our mature muscle cells. We identified 21 transcription factors expressed including a LIM gene (actin-binding LIM), a t-box gene, a SINE class homeobox gene, a SMAD gene, 3 bZIP factors, and a basic helix-loop-helix factor (Additional file 9: Table S2 and GitHub repository). In addition, we found 11 genes associated with developmental signaling including 4 MAPK genes, an FGF receptor, a STAT gene, a nuclear receptor (MlNR2), and more (GitHub repository). Lastly, we identified JAK (ML11617a) expression in three out of four muscle cells and STAT (ML09844a) in all four cells (GitHub repository).

We compared expression profiles of our four muscle cells to a previously published droplet-based single-cell RNA-Seq dataset of adult *Mnemiopsis* cells [33]. That study defined 54 transcriptionally coherent metacell clusters (approximating cell types), each characterized by an average of 132 marker genes. For each metacell, we evaluated the expression of its marker genes in our muscle transcriptomes, considering both mean expression (TPM) and the number of expressing cells. Across these metrics, our data most closely matched the unannotated metacell C29 (mean TPM = 1997; mean count = 3.58). The next closest match was metacell C47 (mean TPM = 1182; mean count = 3.56), which had been annotated as smooth muscle (Additional file 5: Fig. S3).

## Discussion

Our integrated molecular and electrophysiological analyses of *Mnemiopsis* muscle cells reveal a functionally competent, voltage-gated calcium signaling system with molecular and physiological characteristics broadly comparable to those of excitable cells in cnidarians and bilaterians. These findings contribute important data to emergent genomic and transcriptomic resources and provide key insights into the capacity and molecular composition of excitable cells in the last common ancestor of all animals (Fig. 1A).

### Electrophysiological properties of voltage-gated channels in *Mnemiopsis* muscle cells

Our molecular and electrophysiological analyses show that *Mnemiopsis* excitable cells express a basic set of voltage-gated channels (calcium, sodium, and potassium) and can generate and transmit action potentials. The general properties of these electrical currents are consistent with those found in the excitable cells of non-ctenophore animals.

Our genomic analyses of voltage-gated calcium and sodium channels support earlier genomic surveys [39, 40, 47]. *Mnemiopsis* has a single voltage-gated calcium channel gene (ML011744a, Ca_V_2) with high field strength site signature EEEE suggesting high calcium selectivity (e.g., [47]) and two genes coding for voltage-gated sodium channels (ML17059a, ML358826a) characterized by pore motif DEEA suggesting permeability to both Ca^2+^ and Na^+^ ions (e.g., [48]).

*Mnemiopsis* VGSCs exhibit an unusually pronounced window current, indicating that a substantial fraction may remain functional even across a broad range of depolarization. The functional role of these window currents remains uncertain. Although speculative in the context of this study, they may contribute to the resting membrane potential of *Mnemiopsis* excitable cells and contribute to Na^+^ and Ca^2+^ influx underlying excitability and rhythmo-/arrhythmogenesis of sensory and neuronal cells, as well as excitation–contraction coupling in muscle cells. However, the specific roles of these currents can be better understood within defined physiological and molecular contexts, including complex interactions with other sodium and calcium transport systems.

In non-ctenophore animals, muscle contraction is typically triggered by a calcium-dependent mechanism involving synaptic signaling and rapid electrical impulses regulated by voltage-gated channels. Bilaterians typically possess three classes of VGC channels (Ca_V_1, Ca_V_2, and Ca_V_3) with Ca_V_1 and Ca_V_3 channels often playing a role in muscle physiology whereas Ca_V_2 genes are usually confined to neurons. Although the last common metazoan ancestor possessed Ca_V_1, Ca_V_2, and Ca_V_3 genes [49], *Mnemiopsis* and other ctenophores retain only a single Ca_V_2, consistent with the loss of Ca_V_1 and Ca_V_3 in the stem lineage leading to the last common ctenophore ancestor. Our electrophysiology data suggest that *Mnemiopsis* voltage-gated channels play an important role in muscle excitation. Furthermore, we propose that calcium influx mediated by these channels is strongly reinforced by an inward calcium component of predominantly single type of *Mnemiopsis* VGSC, which weakly discriminates between monovalent cations and calcium.

Although our electrophysiological data support the functional expression of voltage-gated calcium channels, VGCC transcripts were only detected in one of our muscle cell transcriptomes. Several factors may explain this apparent discrepancy. VGCCs have long half-lives, and this slow turnover may be associated with extremely low transcript levels. VGCC transcripts can be trafficked to specific subcellular compartments (e.g., remote processes) and translated locally. Lastly, our individual muscle cells were sequenced to varying depths, and VGCC expression was detected only in the cell with the second highest number of expressed genes (see CSV count files in our GitHub repository). It may be that VGCC was expressed at low levels across all muscle cells but was detected only in one.

Intriguingly, in some *Mnemiopsis* muscle cells, we observed that the generation of action potentials or excitation does not always lead to contraction. In other words, there is an excitation range over which muscle fibers can generate and transmit action potentials, accompanied by little, if any, contraction. The ability of muscle cells to generate action potentials without contracting suggests a multimodal or hybrid role for these cells, potentially contributing more substantially to the overall signaling and communication network in *Mnemiopsis*.

Intercellular communication involving muscle cells could be mediated by the activity of innexons through either gap junctional connections or paracrine signaling [38]. Previously we proposed that the muscle cells of *Mnemiopsis* express innexins and exhibit physiological properties consistent with gap junctional channel activity [38]. Here we confirmed the expression of the *Mnemiopsis* innexins INXO (ML036514a, 110.8 ± 69.7) and INXD (ML25999a, 465.3 ± 155.6) in all four single muscle cell samples. While INXD is ubiquitously (or near-ubiquitously) expressed in *Mnemiopsis* cells, INXO appears to be specific to muscle and putative neurons [33, 38]. In bilaterians, muscle cells are often electrically coupled by gap junctions [50]. Our recordings of connected muscle cells (Additional file 6: Fig. S4) show correlated activity between the pair, consistent with coupling via innexin-based gap junctions—an arrangement also observed in the muscle cells of cnidarians and bilaterians.

### Potassium channels

*Mnemiopsis* lack Shab, Shal, and Shaw voltage-gated potassium channels, but have a highly expanded set of Shaker channels [43, 44]. Several Shakers have been expressed heterologously and four of these, MlShak4 (ML10392a), MlShak5 (ML141713a), MlShak13 (ML01366a), and MlShak15 (ML02524b), exhibit pronounced inactivation [45]. MlShak15 is characterized by kinetic and voltage-dependent parameters that align well with the currents shown in Fig. 4. Three of our four sequenced muscle cells express at least one of these genes. The one cell that does not express one of these has a gene expression profile with the least number of detected genes (see CSV files in our GitHub repository).

Activation of BK Ca^2+^-activated K^+^ (KCNMA1) channels in mammalian smooth muscle cells facilitates action potential repolarization and muscle relaxation [51, 52]. The *Mnemiopsis* genome encodes for three KCNMA1 channels (ML04056a; ML128229a; ML01811a), which have very large conductance and are activated by both membrane depolarization and intracellular calcium. Our four muscle cells express at least one of these KCNMA1 genes, with ML128229a being expressed in three of the four cells.

### Exact identification of ion channels in *Mnemiopsis* muscle cells

Identifying the specific ion channels expressed in excitable cells is most accurate when single-cell RNA-Seq is performed on the same cells that were electrophysiologically recorded (Patch-Seq). Here we have recorded and sequenced morphologically similar cells from the same anatomical region. Accordingly, our analyses cannot definitively determine the molecular identity of voltage-gated and other ion channels that generated the currents that we observed. Additional work is required to pinpoint the specific channel proteins that shape ctenophore muscle currents. Nevertheless, our data provide a wealth of insights into the electrophysiology of and will be particularly helpful for predicting the subunit composition of multimeric channel assemblages in future functional studies.

### Calcium signaling and electrophysiology

Recent advancements in imaging techniques combined with powerful computational approaches (machine learning) have provided unprecedented opportunities for studying nervous systems. We have developed a simple method to greatly improve loading *Mnemiopsis* cells with standard cell-permeable (acetoxymethyl (AM) neutralized) fluorescent probes and confirm that the intracellular calcium activity detected in this preparation can be used reliably to estimate the electrophysiological activity of the cell. We have also developed a primary cell culture preparation that maintains the viability of the *Mnemiopsis* cells for many hours (Additional file 2: Fig. S1; Additional file 6: Fig. S4 and Additional file 1: Video 1; Additional file 3: Video S1; Additional file 7: Video 2; Additional file 8: Video 3).

This advancement promotes the ability to screen for cells sensitive to potential neurotransmitters or neuromodulators, perform targeted pharmacological tests with receptor/channel agonists/antagonists, and study the molecular mechanisms of signal transduction in great detail, potentially including the role of intracellular compartments, involvement of either ionotropic or metabotropic signaling [53], and calcium puffs localized to active gap-junction channel zones [38]. After identifying cells or cell types in imaging experiments, they can be further analyzed using electrophysiological and transcriptome techniques. This can help determine whether *Mnemiopsis* excitable cells have specific combinations of voltage-dependent and/or voltage-independent conductances that are known to be important for generating sensory responses and mediating action potentials. The data obtained from single-cell imaging experiments can also serve as a foundation for future studies that involve selectively blocking specific conductances using pharmacological agents. This approach can help implicate these conductances in the activity of neural circuits.

### Single muscle cell transcriptome analysis and molecular profile of muscle cells

While the primary goal of the single muscle transcriptome analysis was to identify genes underlying the electrophysiological properties of these cells, we also investigated the molecular profile underlying muscle cell specification. In differentiated cells, it is common for a subset of cell-type specification genes to be expressed continuously to maintain cell identities (e.g., [54, 55]). This continuous expression of terminal selector genes (reviewed in [56]) offers insight into the specification of ctenophore muscle cells.

Among the transcription factors detected in all muscle cells, we identified a JUN-related transcription factor (AP-1) and a CREB3 ortholog (cyclic AMP-responsive element-binding protein, CREB). We also found that bZIP-domain-containing ATF6 (cyclic AMP-dependent transcription factor ATF-2) is highly expressed in all four muscle cells. This factor is a known target of p38 MAPK signaling, which is essential for myogenesis in bilaterians [57], suggesting a potential role in cell cycle exit and muscle differentiation in ctenophores [58]. We also detected MEF2, another target of MAPK signaling and a major regulator of muscle differentiation in bilaterians, in two of the four muscle cells. The function of MEF2 in ctenophores is unclear, but its expression suggests that, as in non-ctenophores, it may play a key role in muscle development. We also detected components of the JAK-STAT signaling pathway including ML11617a, a putative JAK1/2/3 gene, expressed in three out of four muscle cells. The JAK-STAT pathway plays a key role in muscle differentiation in non-ctenophores [59]. Many of the developmental genes we identified in ctenophore muscles have roles in specifying the muscle cells of non-ctenophores.

## Conclusions

Our integrative analysis of electrophysiological, calcium imaging, and single-cell transcriptomic data from *Mnemiopsis* muscle cells provides a comprehensive view of how excitable cells operate in ctenophores—a key lineage for reconstructing the last common ancestor of animals. We demonstrate that ctenophore muscle cells possess functional voltage-gated calcium, sodium, and potassium channels, exhibit calcium activity consistent with their electrophysiological profile, and express a distinct but recognizable complement of ion channel and signaling genes also found in other animals. These findings support the idea that ctenophore excitable cells are capable of complex electrical and calcium signaling but also suggest that the molecular and physiological toolkit for excitability was already established in the last common ancestor of extant animals. By bridging cellular physiology with molecular identity, our work offers key insights into the early evolution of neural and muscular systems and provides a novel contribution towards reconstructing the fundamental properties of electrical cell types in the last common ancestor of animals.

## Methods

### *Mnemiopsis* specimens

*Mnemiopsis* specimens were collected from the intracoastal waterway, near the Whitney Laboratory for Marine Bioscience in St. Augustine, FL. In some cases, specimens were analyzed soon after being collected and other times they were maintained in Kriesel chambers [36, 59] on a diet of brine shrimp (*Artemia salina*) and/or rotifers (*Brachionus plicatilis*).

### Cell culture

To isolate muscle cells, small sections of *Mnemiopsis* lobes containing muscle cell layers and mesoglea were mechanically triturated in 400–500 µL in modified artificial sea water (ASW, see Solutions). In some cases, hyaluronidase (1 mg/ml, Sigma-Aldrich, H3506) and collagenase (collagenase type I, 1 mg/ml, Sigma-Aldrich, SCR103) were used to isolate muscle cells from the surrounding mesoglea. Dissociated cells were plated on 35-mm Petri dishes filled with ASW and allowed to settle for at least 1 h before recording. Dishes were kept at room temperature and used within 2–3 h. Smooth muscle cells were identified based on morphology—elongated shape with multiple processes (Fig. 2)—and function, including spontaneous or potassium-evoked contractions. High-potassium intracellular solution (see Solutions) was applied focally through the recording pipette to test excitability. For our experiments, we typically selected cells that lacked visible contacts with neighboring cells. Shortened muscle fiber segments (50–300 mm) were selected to improve space clamp during whole-cell recordings.

### Electrophysiological recording and analysis

Patch electrodes for extracellular (spikes) and whole-cell recordings were pulled from borosilicate capillary glass (Sutter Instrument, BF150-86-10) using a Flaming-Brown micropipette puller (P-87, Sutter Instruments). All currents were recorded using an Axopatch or 200B amplifier controlled by Digidata 1322 A, 1440 A and pClamp 9.2–10.7 and data sampled at 5 kHz. Action potentials (Aps, spikes) were recorded in track mode using cell-attached loose-patch configuration. Whole-cell currents were recorded in voltage-clamp mode (amplifier configuration—whole cell *b* = 0.1) at a holding potential of − 70 mV unless otherwise noted. Bath solution change or neurotransmitter candidate application for both extracellular AP and whole-cell recordings was performed with a rapid solution changer, RSC-160 (BioLogic Science Instruments, France). Experiments were carried out at ~ 21 °C. Data were analyzed with Clampfit 9.2–10.7 (Molecular Devices) in combination with SigmaPlot 14 (SPSS Inc., Chicago, IL, USA). All results are expressed as means ± SEM of *n* observations.

### Calcium imaging

Calcium imaging was performed using either Fluo-4 AM (Thermo Fisher Scientific, Waltham, MA, USA; cat. No. F14201) or Calbryte™ 520 AM (AAT Bioquest, Inc., Pleasanton, CA, USA; cat. No. 20650). Dyes were prepared in artificial sea water containing 0.04% (w/v) Pluronic F-127 and applied at a final concentration of 5 µM for ~ 30 min at room temperature. Following incubation, dissected *Mnemiopsis* cells were rinsed in dye-free ASW and placed on the stage of an inverted microscope (Olympus IX-71, Olympus Corp., Tokyo, Japan) equipped with a cooled CCD camera (ORCA R2, Hamamatsu, Hamamatsu City, Japan).

For simultaneous recordings of electrophysiological and calcium signals, muscle cells were injected with a calcium-sensitive probe (Fluo4 penta-potassium salt, ~ 5 μM, whole-cell intracellular pipette solution) through a patch pipette.

Fluorescence imaging was performed under the control of Imaging Workbench 6 software (INDEC Systems). A standard FITC filter set (excitation at 510 nm, emission at 530 nm) was used for single-wavelength measurements. Images were collected usually at the rate ∼3, 5, or 24 Hz (specified in the figure legends). Stored time series image stacks were analyzed offline using Imaging Workbench 6, Clampfit 9.2–10.7, SigmaPlot 10/14 or exported as TIFF files into ImageJ 1.42–1.53 (http://rsbweb.nih.gov/ij/index.html).

### Solutions and chemicals

For whole-cell recordings, *Mnemiopsis* muscle cells were bathed in artificial sea water (ASW) containing (in mM): 460 NaCl, 10–20 KCl, 11 CaCl_2_, 55 MgCl_2_, 10 HEPES. In some experiments, modified saline solutions were used: (1) low-magnesium sea water containing (in mM) 486 NaCl, 10–20 KCl, 13.6 CaCl_2_, 9.8 MgCl_2_, and 10 HEPES; (2) zero-magnesium seawater containing (in mM) 486 NaCl, 5 KCl, 13.6 CaCl_2_, 30 glucose, and 10 HEPES; and (3) divalent-free seawater containing (in mM): 486 NaCl, 20 KCl, 70 glucose, 10 HEPES, 1 EGTA, and 1 EDTA). Pipette electrodes used for extracellular recordings were filled with artificial sea water.

The whole-cell pipette solution contained (in mM): 210 KCl, 0–0.1 CaCl_2_, 1–2 EGTA, 696 glucose, and 10 HEPES. For calcium imaging experiments, the intracellular solution contained (in mM): 210 KCl, 0.5 GTP, 0.5 ATP, 1 MgCl_2_, 696 glucose, 1 EGTA, and 10 Hepes. In some experiments, KCl was replaced with CsCl (210 mM). All solutions were adjusted to pH 7.7–7.9 using NaOH or Trizma base (Sigma-Aldrich). Inorganic salts were purchased from Thermo Fisher Scientific, and organic compounds from Sigma-Aldrich. Free Ca^2+^ concentrations were estimated using WebmaxC (https://somapp.ucdmc.ucdavis.edu/pharmacology/bers/maxchelator/webmaxc/webmaxcS.htm).

### Single-cell collection and scRNAseq analysis

*Mnemiopsis* cells were isolated and maintained in primary cell culture as described above. Single muscle cells were identified based on morphology and collected using disposable glass suction pipettes. Pipettes were fabricated from borosilicate capillary glass (BF150-86-10, Sutter Instrument, CA, USA) using a Flaming-Brown micropipette puller (P-87, Sutter Instrument, CA, USA). The diameter of the pipettes varied, but typically did not exceed 7 µm. Larger diameters were used for collecting larger cells. The pipette solution contained (in mM): 210 NaCl, 696 glucose, 10 HEPES, and 1 EGTA, adjusted to 7.8 pH.

Once a cell was captured, it was transferred to 3–4 µl of lysis buffer. The sample was then sonicated for ~ 5 s in ice-cold water and immediately stored on dry ice. The lysis buffer contained nuclease free water, 3.3 mM dNTPs, 1.33 U/µL Rnase inhibitor, and 0.133% Triton X-100. All reagents were obtained from Thermo Fisher Scientific (MA, USA). Samples were stored at − 80 °C until library preparation and sequencing.

### Transparency and reproducibility

Commands, scripts, and data files used in these analyses are available at our GitHub repository [60].

### Transcriptome analysis

We used Bowtie2 [61] and RSEM [62] as implemented in the utility script align_and_estimate_abundance.pl distributed with Trinity (v2.8.5; [63]) to align reads from muscle cells to ML2.2 *Mnemiopsis* gene models (v1), generate counts, and normalize those counts (normalized as transcripts per million, TPM). Command lines and information required to replicate these analyses are in our GitHub repository [60].

### Trinotate

We used Trinotate [64] to annotate the *Mnemiopsis* protein models (ML2.2). This allowed us to generate improved gene ontology annotations for ML2.2. We followed the steps outlined in the Trinotate Functional Annotation tutorial [65] (https://github.com/griffithlab/rnaseq_tutorial). Briefly, we BLASTed ML2.2 protein models vs Swissprot [66] and ran a Hmmer hmmscan search [67] against PFAM [68] and then used the Trinotate script to generate an annotation file. Command lines, resulting data file, and information required to replicate these analyses are in our GitHub repository [60].

### Genes expressed in all four muscle cells

We identified 1616 ML2.2 gene models expressed in all four muscle cells. We did not use a threshold as these transcriptomes were of isolated and washed cells and therefore, we expect that every read came from the cell that was sequenced. This is unlike bulk RNA-Seq, where non-target cells are almost always included, and droplet-based single-cell sequencing, where ambient RNA can contaminate individual cells. The list of these genes and their corresponding transcript-per-million (TPM) values are available in our GitHub repository [60].

### Gene ontology analyses

We used a Monte Carlo analysis to identify gene ontology categories (biological process, cellular component, and molecular function) that were overrepresented in the 1616 gene models expressed in all four muscle cells. This approach was applied in Babonis et al. [69]. Command lines and data files required to replicate these analyses are in our GitHub repository [60].

## Supplementary Information

Additional file 1: Video 1. Calcium imaging of a spontaneously active muscle cell in primary culture. The recording duration is approximately 1 min. Acquisition rate: 5 fps. The video is shown at 10 × speed (50 fps). Scale bar: 20 µm.

Additional file 2: Fig. S1. Ligand-evoked electrophysiological activity of isolated ctenophore *Mnemiopsis* muscle cells. Responses of ctenophore muscle cells to pulses of a potential neuromuscular transmitter application (blue bar) were recorded using loose-patch clamp configuration. These cells are sensitive to glutamate and capable of generating action potentials in a consistent manner. Multi-channel rapid solution changer (RSC-160, BioLogic) was used for neurotransmitter/s application. Simultaneous video recording (Video S1) shows evoked contractile activity of the cell. GLU = glutamate; L-SER = l-serine; GLY = glycine; D-SER = d-serine. The ligands were applied at a maximum concentration of 1 mM; however, 500 ms pulses delivered the ligands at lower concentration. Scale bar 20 μm. See Additional file 3: Video S1 for corresponding video.

Additional file 3: Video S1. Evoked contractile activity of a muscle cell. The recording duration is approximately 5.5 min. Acquisition rate: 5 fps. The video is shown at 10 × speed (50 fps). Scale bar: 20 μm.

Additional file 4: Fig. S2. Muscle cells collected for single-cell RNA sequencing. (A) Lobe region showing multiple cell types within mesoglea including muscle fibers. (B) Calcium image of the same region. (C) Muscle cell 3a isolated for sequencing (SRA accession = SRR35950088). (D) Muscle cell 1a isolated for sequencing (SRA accession = SRR35950089). (E) Muscle cell 5a isolated for sequencing (SRA accession = SRR35950087). (F) Muscle cell 6a isolated for sequencing (SRA accession = SRR35950086). Scale bars: 150 μm (A–B); 20 μm (C–F).

Additional file 5: Fig. S3. Muscle single-cell transcriptomes compared to Sebé-Pedrós et al. [33] droplet-based single-cell metaclusters. The scatter plot shows all 54 metacell clusters from Sebé-Pedrós et al. [33], with average TPM on the x-axis and average count on the y-axis. The maximum count would be 4 in which every marker gene from a particular metacell would be expressed in all 4 of the muscle cells. The 10 cells exceeding 500 average TPM are highlighted in coral and labeled: C45, C6, C4, C3, C22, C44, C46, C2, C47, and C29—with C29 being the highest at nearly 2000 TPM. C45, C46, and C47 were labeled as smooth muscle in Sebé-Pedrós et al. [33] and C29 was unlabeled. C53 was labeled as a striated muscle in Sebé-Pedrós et al. [33].

Additional file 6: Fig. S4. Coordinated electrical and calcium activity in primary muscle cells. (A) Two representative muscle cells loaded with the calcium indicator Fluo-4 during simultaneous extracellular loose patch recording (recording pipette shown). (B) Spontaneous electrical, spiking, activity of the individual muscle cell (red). (C) Corresponding calcium signal recordings from the same cell (red trace) reveal that generation of bursts of action potentials coincides with increase in intracellular calcium concentration. Note that calcium signals (C, red and blue traces) recorded from two adjacent cells correlate, suggesting either paracrine and/or electrical-based intercellular signaling. Scale bar 20 µm.

Additional file 7: Video 2. Calcium imaging of a spontaneously active putative neuron in primary culture. The recording duration is approximately 1 min. Acquisition rate: 5 fps. The video is shown at 10 × speed (50 fps). Scale bar: 20 μm.

Additional file 8: Video 3. Calcium imaging of a spontaneously active, putative epithelial cell in primary culture. The recording duration is approximately 1 min. Acquisition rate: 5 fps. The video is shown at 10 × speed (50fps). Scale bar: 20 µm.

Additional file 9: Table S1. Muscle cell expression of genes belonging to muscle-associated GO categories. Table S2. Muscle cell expression of channels, transcription factors, and signaling molecules with potential roles in bilaterian muscle cells. * indicates MLID also appears in Table S1. ** indicates expression in 3 of 4 cells.

## Data Availability

The transcriptomic data generated in this study can be found in NCBI’s Short Read Archive (Bioproject accession: PRJNA1357847) [70]. Code and other data files are available in GitHub (https://github.com/josephryan/Mnemiopsis_muscle). An archive of the repo at the time of publication is available under the DOI (https://doi.org/10.5281/zenodo.17523955).

## Abbreviations

VGC: Voltage-gated channels
VGSC: Voltage-gated sodium channels
VGCC: Voltage-gated calcium channels
scRNA-Seq: Single-cell ribonucleic acid sequencing
GO: Gene ontology
TPM: Transcripts per million
CSV: Comma separated values
ASW: Artificial sea water
