# Supplementary material for: Molecular and electrophysiological properties underlying calcium signaling in muscle cells of ctenophores
Source: BMC Biol. 2026 Aug 13;24:232. doi: 10.1186/s12915-026-02705-5 (PMC13749725; doi:10.1186/s12915-026-02705-5)
Supplement: Supplementary file 1 [file 12915_2026_2705_MOESM2_ESM.pdf]

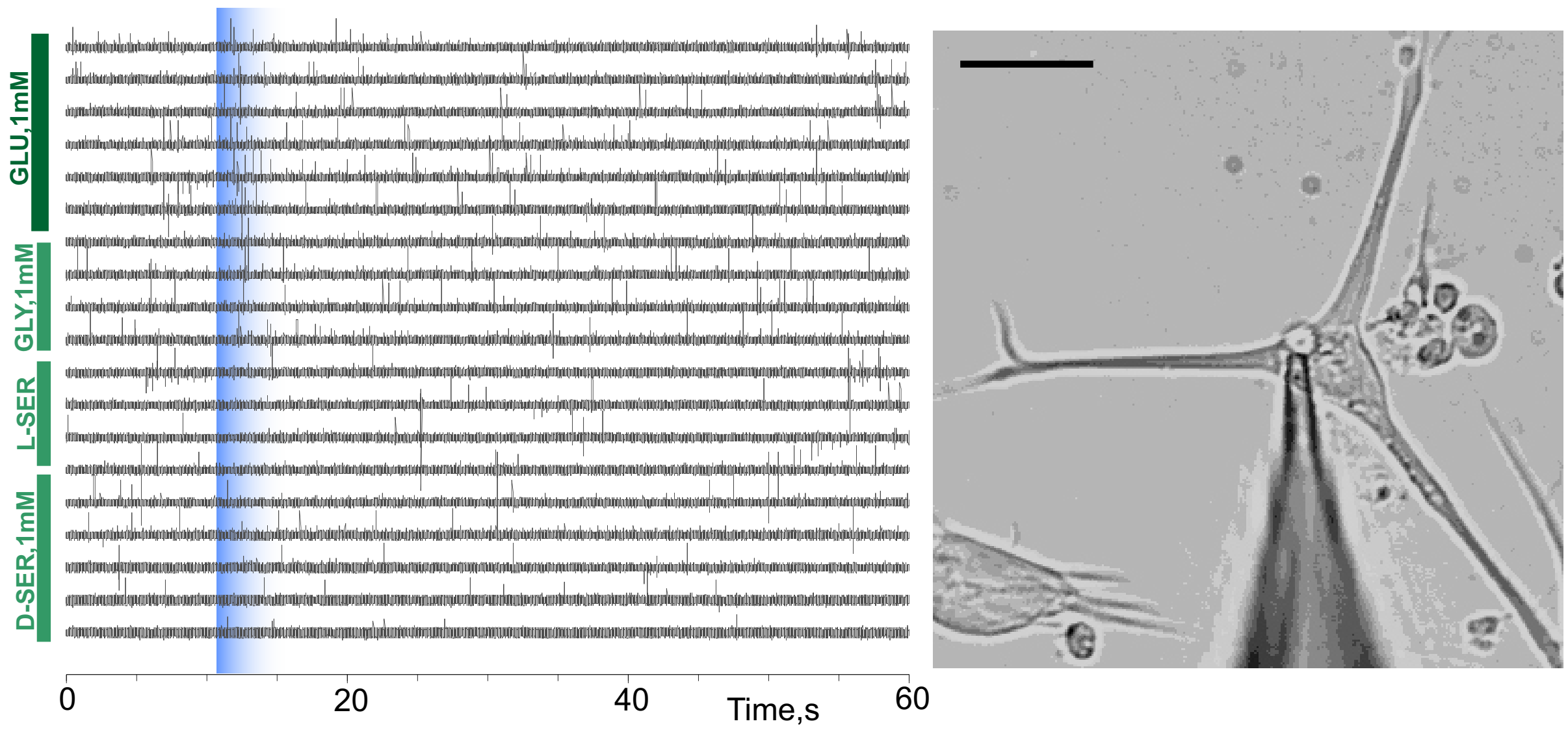

GLU,1mM
GLY,1mM
L-SER
D-SER,1mM
0
20
40
60
Time,s
