# Supplementary material for: Molecular and electrophysiological properties underlying calcium signaling in muscle cells of ctenophores
Source: BMC Biol. 2026 Aug 13;24:232. doi: 10.1186/s12915-026-02705-5 (PMC13749725; doi:10.1186/s12915-026-02705-5)
Supplement: Supplementary file 5 [file 12915_2026_2705_MOESM9_ESM.docx]

**Additional file 9: Table S1. Muscle cell expression of genes belonging to muscle-associated GO categories**

| **MLID** | **AVG TPM** | **TRINOTATE ANNOTATION** | **GO CATEGORIES** | |
| --- | --- | --- | --- | --- |
| **CELLULAR COMPONENT** | | | |  |
| ML03977a | 7009.2 | Myosin regulatory light chain 12B | Z disc (GO:0030018) | |
| ML04011a | 1705.3 | Heat shock protein HSP 90-alpha |  |  |
| ML13041a | 1069.2 | Myosin regulatory light chain 12B |  |  |
| ML03394a | 791.1 | Myosin regulatory light chain 12B |  |  |
| ML02959a | 647.7 | LIM, Cysteine-rich protein 2 (CRP in Koch et al. 2012) |  |  |
| ML04967a | 184.2 | Alpha-parvin |  |  |
| ML271531a | 141.5 | F-actin-capping protein subunit beta |  |  |
| ML21021a | 63.7 | Ankyrin-1 |  |  |
| ML077615a | 36.6 | Serine/threonine-protein kinase PAK 2 |  |  |
| ML06706a | 24.7 | Filamin-B |  |  |
| ML131135a | 12.4 | Sodium/calcium exchanger 3 |  |  |
| ML00084a | 7.7 | FH1/FH2 domain-containing protein 3 |  |  |
| ML018044a | 1.2 | Filamin-B |  |  |
| ML13538a | 222.5 | Proteasome subunit alpha type-6 | Myofibril (GO:0030016) | |
| ML022011a | 97.1 | Myosin heavy chain |  |  |
| ML02275a | 71.2 | Myosin heavy chain |  |  |
| ML070220a | 268.5 | Fructose-bisphosphate aldolase A | I band (GO:0031674) | |
| ML01103a | 36.4 | Protein phosphatase 1 regulatory subunit | Contractile fiber (GO:0043292) | |
| **BIOLOGICAL PROCESS** | | | |  |
| ML08042a | 326.0 | Tropomodulin-1 | Muscle contraction (GO:0006936) | |
| ML046417a | 41.5 | T-box transcription factor TBX20 |  |  |
| ML063321a | 12.5 | Titin |  |  |
| ML05198a | 154.4 | Laminin subunit alpha-2 | Muscle organ development (GO:0007517) | |
| ML148524a | 149.2 | Collagen type IV alpha-3-binding protein |  |  |
| ML20029a | 61.1 | Transcriptional enhancer factor TEF-3 |  |  |
| ML05951a | 14.7 | Laminin subunit gamma-1 |  |  |
| ML064954a | 21.4 | Sine-class Homeobox (SIX27 in Ryan et al. 2010) | Striated muscle cell differentiation (GO:0051146) | |
| **MOLECULAR FUNCTION** | | | |  |
| ML00303a | 1356.4 | Coactosin | Actin filament binding (GO:0051015) | |
| ML14123a | 525.4 | Plastin-2 |  |  |
| ML14271a | 266.5 | Allograft inflammatory factor 1 |  |  |
| ML09179a | 193.0 | Coronin-1B |  |  |
| ML38815a | 97.3 | Supervillin |  |  |
| ML218949a | 35.6 | Alpha-adducin |  |  |
| ML03212a | 27.1 | Abelson tyrosine-protein kinase 2 |  |  |
| ML38816a | 20.3 | Supervillin |  |  |
| ML19986a | 1247.1 | Dynein light chain 2 | Cytoskeletal protein binding (GO:0008092) | |
| ML19987a | 737.6 | Dynein light chain 2 |  |  |
| ML068319a | 218.3 | Gelsolin-like protein 1 | Actin binding (GO:0003779) | |
| ML04674a | 130.4 | Actin-binding LIM protein 1 |  |  |
| ML05655a | 108.5 | Filamin-A |  |  |
| ML093519b | 54.3 | LIM, Actin-binding LIM protein (ABLIM class; Koch et al. 2012) |  |  |
| ML033628a | 52.9 | WD repeat-containing protein 1 |  |  |
| ML131118a | 29.5 | Protein enabled |  |  |
| ML08716b | 23.0 | Protein-methionine sulfoxide oxidase mical2b |  |  |
| ML08503a | 22.4 | Bud site selection protein 6 |  |  |
| ML00266a | 12.2 | Unconventional myosin-If |  |  |
| ML334211a | 6.3 | Dematin |  |  |
| ML07344a | 5.4 | Alpha-ketoglutarate-dependent dioxygenase alkB |  |  |

**Additional file 9: Table S2. Muscle cell expression of channels, transcription factors, and signaling molecules with potential roles in bilaterian muscle cells.**

* Indicates MLID also appears in Table S1. ** Indicates expression in 3 of 4 cells

| **MLID** | **AVG TPM** | **ANNOTATION** |
| --- | --- | --- |
| **CHANNELS/RECEPTORS** | | |
| ML26173a | 1248.6 | voltage-dependent anion-selective channel |
| ML25999a | 465.3 | innexin (INXD) |
| ML017941a | 128.6 | chloride intracellular channel protein |
| ML032110a | 124.4 | amiloride-sensitive cation channel |
| ML036514a | 110.8 | innexin (INXO) |
| ML00254a | 80.8 | adhesion G protein-coupled receptor L3 |
| ML17563a | 77.6 | voltage-gated K+ channel |
| ML04825a | 54.6 | sodium channel protein type 2 subunit alpha |
| ML01419a | 32.0 | neuronal pentraxin receptor |
| ML047929a | 31.2 | neuropeptide FF receptor 2 |
| ML03598a | 30.9 | voltage-gated potassium channel subunit beta-2 |
| ML172117a | 25.7 | TMEM38A trimeric intracellular cation channel type A |
| ML051723a | 17.1 | two-pore calcium channel |
| ML07344a | 5.4 | glutamate receptor |
| **TRANSCRIPTION FACTORS** | | |
| ML066516a** | 111.6 | bZip, TFE3, transcription factor E3 |
| ML154140a | 97.5 | MADS box, SRF, Serum response factor |
| ML046417a | 41.5 | T-box, T-box transcription factor TBX20 |
| ML064954a* | 21.4 | SIX, Sine-class Homeobox protein (SIX27 in Ryan et al. 2010) |
| ML278913a** | 17.2 | Myocardin related transcription factor |
| ML31952a** | 1.1 | bHLH, CLOCK, clock circadian regulator |
| **OTHER MUSCLE-RELATED GENES** | | |
| ML04674a | 130.4 | LIM, Actin-binding LIM protein (ABLIM class; Koch et al. 2012) |
| ML093519b | 54.3 | LIM, Actin-binding LIM protein (ABLIM class; Koch et al. 2012) |
| ML063322a** | 41.1 | Myosin light chain kinase, smooth muscle |
| ML108023b** | 8.4 | ENIGMA (ZASP related in Koch et al. 2012) |
